# Affect and Subsequent Physical Activity: An Ambulatory Assessment Study Examining the Affect-Activity Association in a Real-Life Context

**DOI:** 10.3389/fpsyg.2016.00677

**Published:** 2016-05-09

**Authors:** Christina Y. N. Niermann, Christian Herrmann, Birte von Haaren, Dave van Kann, Alexander Woll

**Affiliations:** ^1^Institute of Sports and Sports Science, Karlsruhe Institute of TechnologyKarlsruhe, Germany; ^2^Department of Sport, Exercise and Health, University of BaselBasel, Switzerland; ^3^Institute of Psychology, German Sport University CologneCologne, Germany; ^4^Department of Health Promotion, Maastricht UniversityMaastricht, Netherlands

**Keywords:** multilevel regression model, accelerometer, ecological momentary assessment, diary, habit, mood

## Abstract

Traditionally, cognitive, motivational, and volitional determinants have been used to explain and predict health behaviors such as physical activity. Recently, the role of affect in influencing and regulating health behaviors received more attention. Affects as internal cues may automatically activate unconscious processes of behavior regulation. The aim of our study was to examine the association between affect and physical activity in daily life. In addition, we studied the influence of the habit of being physically active on this relationship. An ambulatory assessment study in 89 persons (33.7% male, 25 to 65 years, *M* = 45.2, *SD* = 8.1) was conducted. Affect was assessed in the afternoon on 5 weekdays using smartphones. Physical activity was measured continuously objectively using accelerometers and subjectively using smartphones in the evening. Habit strength was assessed at the beginning of the diary period. The outcomes were objectively and subjectively measured moderate-to-vigorous physical activity (MVPA) performed after work. Multilevel regression models were used to analyze the association between affect and after work MVPA. In addition, the cross-level interaction of habit strength and affect on after work MVPA was tested. Positive affect was positively related to objectively measured and self-reported after work MVPA: the greater the positive affect the more time persons subsequently spent on MVPA. An inverse relationship was found for negative affect: the greater the negative affect the less time persons spent on MVPA. The cross-level interaction effect was significant only for objectively measured MVPA. A strong habit seems to strengthen both the positive influence of positive affect and the negative influence of negative affect. The results of this study confirm previous results and indicate that affect plays an important role for the regulation of physical activity behavior in daily life. The results for positive affect were consistent. However, in contrast to previous reports of no or an inverse association, negative affect decreased subsequent MVPA. These inconsistencies may be—in part—explained by the different measurements of affect in our and other studies. Therefore, further research is warranted to gain more insight into the association between affect and physical activity.

## Introduction

Explaining and predicting health behavior and health behavior change has long been an important topic in health psychological research, and several theoretical models and approaches have been developed and used (Rothman, 2000; Schwarzer, 2008). Most models assume that health behaviors such as physical activity are consciously regulated by cognitive, motivational, and volitional processes (Gibbons et al., 2009). These commonly used models only modestly explain and predict health behavior (Williams, 2008; Rhodes and Yao, 2015). Recently, several authors have introduced new perspectives such as unconscious influences on behavior and the role of affect (Williams, 2008; Schwarzer, 2014; Sniehotta et al., 2014). Health behaviors such as physical activity and eating are behaviors of daily life, which are not necessarily regulated by conscious processes such as planning (Sheeran et al., 2013). Everyday life health behaviors are regulated in two ways (Kremers et al., 2006): via cognitive, motivational, and volitional processes and via processes triggered automatically by external (situational or environmental) features or internal cues (affects or emotions; Bargh and Chartrand, 1999; Slovic et al., 2005). According to Russell (2003) affect is conceptualized as “a neurophysiological state that is consciously accessible as a simple, nonreflective feeling” (p. 147). Other terms used for describing this concept are “mood”or “feeling” (Russell, 2003). Affects play an important role in regulating human behavior and influence behavioral reflexes, motivational processes underlying various behaviors as well as complex decision making (Russell, 2003; Naqvi et al., 2006). Automaticity of behavioral reactions is adaptive and beneficial, and humans would not be able to accomplish daily life if even simple decisions and actions would involve deliberate thoughts (Bargh and Chartrand, 1999; Chartrand and Bargh, 1999). Habits represent such an automated pathway to action (Gardner, 2014).

### Affect and physical activity

Although several studies have examined the relationship between affect and physical activity (Williams, 2008), affective processes preceding behavior have received less attention in health psychological research than cognitive, social, and environmental determinants. Exercise, affective response, and exercise adherence are presumably part of a causative chain. While to date the link between affective response and adherence has received little attention, many studies have examined the link between exercise and affective responses. Overall, these studies have shown that physical activity influences positive (but not negative) affect, and engaging in physical activities has psychological benefits (Schwerdtfeger et al., 2010). However, this relationship is not that simple, it depends on intensity and duration of physical activity (Ekkekakis, 2005).

Most previous studies have focused on the unidirectional relationship between affect and physical activity. Considering the role of affects as internal cues triggering automatic processes of behavior regulation suggests the possibility that the relationship between affect and physical activity is bidirectional. However, to date only few studies have investigated affect as predictor of subsequent physical activity (Schwerdtfeger et al., 2010; Liao et al., 2015). Overall, higher positive affect appears to predict higher subsequent physical activity levels but the extent of negative affect does not seem to predict the subsequent physical activity level. Only one study revealed a significant association of negative affect and subsequent physical activity (Dunton et al., 2009).

### Habit and physical activity

A habit is defined as a learned sequence of situational cues that activate a specific goal or end-state and prompt a behavior (Verplanken and Orbell, 2003; Gardner, 2014). Core components are cue-dependence, automaticity, and conditioned stimulus-response association (Orbell and Verplanken, 2010). Thus, habit reflects the impulsive and automatically triggered pathway to behavior. Performing habitual behavior requires little awareness, consciousness, and cognitive effort and is hence highly efficient (Verplanken and Orbell, 2003). Moreover, habits persist even if conscious motivation wanes (Gardner, 2014). In the health behavior context habits have an ambivalent character. Habitual behavior is stable and resistant against “disturbances” which is positive for healthful but negative for unhealthful behaviors, in terms of behavior change interventions (Neal et al., 2006; Verplanken and Wood, 2006; Orbell and Verplanken, 2010). A strong habit to exercise regularly reflects that this behavior is automatically triggered via internal or external cues. Little awareness and cognitive effort is necessary to perform physical activities which makes it less vulnerable to situational disturbing internal or external conditions.

Previous studies have shown that habit strength is related to health behaviors such as healthy diet or physical activity. A systematic review and meta-analysis revealed medium to large effect sizes for the correlation between physical activity habits and physical activity behavior (Gardner et al., 2011). Most studies concentrated on exercise, sedentary behavior, and active commuting, and only few studies focused on overall physical activity (Gardner et al., 2011; Thurn et al., 2014). Moreover, most observational and correlational studies have used subjective measures of physical activity compared to few longitudinal or experimental studies using objective measures of physical activity (Gardner, 2014; Thurn et al., 2014).

Considering the importance of habits and affect in behavior regulation in daily life, these constructs should be examined in a real-life context. Hence, an ambulatory assessment study may be appropriate for studying within person associations between habit strength, affect, and physical activity.

### Ambulatory assessment

To address the relationship between affect and physical activity the characteristics of these complex constructs must be considered and their interplay during short time intervals in daily life must be understood (Shiffman et al., 2008). Affective states underlie dynamic fluctuations across days and even within-days and may be influenced by situational and environmental factors (Stone et al., 1996). Studies using single, retrospective self-reported ratings of affect did not address this association adequately because of limitations such as recall bias (Shiffman et al., 2008). Ambulatory assessment of people in their natural environments can be performed using different methods including momentary self-reports, ecological momentary assessments, and observational and physiological methods (Trull and Ebner-Priemer, 2013). These methods have several advantages: affective states within persons during a week in the participants' natural environment can be assessed repeatedly using electronic diaries, and physical activity and self-reported affective states can be measured simultaneously with accelerometers and mobile devices, respectively. Hence, these methods allow analyzing the interrelationship between affect and physical activity in daily life.

### Aims and hypotheses

The first aim of this study was to examine the association between positive and negative affect and subsequent moderate-to-vigorous physical activity (MVPA) in a real-life context. Ambulatory assessment was used to assess participants' MVPA and affect in their natural environment. We used electronic diaries to assess affective states and self-reported MVPA repeatedly within persons and accelerometers to objectively monitor MVPA. We hypothesized that positive affect is positively related to subsequent MVPA while negative affect is negatively related to subsequent MVPA. The second aim was to investigate the effect of habit strength on MVPA in daily life. We hypothesized that a strong habit to be physically active is associated with more time in MVPA during weekdays. Moreover, we assumed that habitual physical activity could protect this behavior against barriers or low self-control resources (Neal et al., 2013). Therefore, we hypothesized that a strong habit to perform regular MVPA moderates the effect of positive and negative affect on MVPA in the following way: Perceiving a strong habit to exercise regularly should strengthen the effect of positive affective states and should buffer the effect of a negative mood.

## Materials and methods

This ambulatory assessment study was conducted as a part of the multidisciplinary project *EATMOTIVE* funded by the German Federal Ministry of Education and Research. The participants answered questions regarding their physical activity, sedentary behavior, nutrition, mood, daily hassles and conflicts, and family life on a day level using electronic diaries. Additionally, all participants wore an accelerometer to objectively measure their daily physical activity.

### Procedure

We focused on employed adults aged 25–65 years living in the region of Konstanz, Germany. Participants were recruited by initial contact through the “Konstanz Life Study” (Renner et al., 2012) and at two local sport events (games of the regional handball and basketball teams) by the study staff. Eligible participants received verbal information and an information flyer. By writing down their email addresses they agreed to receive an email from the study staff. In addition, university employees received an email with the study information and the option of replying to the email when interested. Interested persons were invited to participate in one of 15 introductory events held every 3 weeks at the University of Konstanz informing participants about the content and the goals of the study. Participants received a smartphone and an accelerometer, learned how to handle the devices and received a paper-pencil questionnaire. Written informed consent was obtained from all participants.

### Ethics statement

The study protocol was defined by a multidisciplinary expert panel of scientists involved in the EATMOTIVE project. The study fully conformed to the Declaration of Helsinki and the ethics guidelines of the German Psychological Society and was approved by the ethics committee of the University of Konstanz. Participants received detailed information regarding voluntary participation, completing questionnaires, and processing of their data according to the ethics guidelines of the German Psychological Society (Deutsche Gesellschaft für Psychologie)^1^.

### Study protocol

On the day prior to the diary period all participants were informed on the study and the measuring devices and completed a paper-pencil questionnaire assessing time-invariant variables such as demographics and habit strength.

Each participant received a smartphone (Samsung GT-I9001) to answer daily questionnaires assessing time-variant variables (e.g., positive and negative affect; MVPA) and an ActiGraph GT3X+ accelerometer (ActiGraph, Pensacola, USA) for assessing physical activity. Each participant was tracked for 7 consecutive days starting on a Wednesday. To measure affect, participants received a short text message including a link to an online questionnaire at fixed times during the day: in the morning (t1, prompted at 6:30 a.m.) to be completed after getting up, in the afternoon (t2, prompted 4:30 p.m.) after finishing work, and in the evening (t3, prompted at 9:00 p.m.) to be completed before going to bed. On weekend days, the morning questionnaire was prompted at 8:00 a.m., and no afternoon questionnaire was prompted. Overall, each participants had to answer 19 daily questionnaires (7 mornings, 5 afternoons, and 7 evenings), but only the 5 work days were used for analyses. Ninety-one persons participated in this study. The participant compliance during the 7 days of the study was high with 97.7% completed questionnaires and 79 participants (85.8%) providing complete data on all days.

### Measures

#### Affect

Despite the advantages of electronic diaries for studying psychological processes and their variability in daily life, these methods are demanding for participants. Hence, these questionnaires should be as short as possible to reduce participants' burden and achieve good compliance (Cranford et al., 2006). As we were interested whether positive and negative affective states predict subsequent MVPA differently, we used an instrument that allows to measure several discrete affects (Gray and Watson, 2007). Therefore, in line with previous studies we used a categorical measure of distinct affective states, the Profile of Mood States-15 (POMS-15; Cranford et al., 2006). We used a German translation of the two subscales, “vigor” and “fatigue,” that represent positive and negative affective states after work (t2). Positive affect represents the experience of a positive mood and negative affect reflects experiencing a negative or aversive mood (Watson and Clark, 1997). Both scales comprise three items (vigor representing a positive affective state: vigorous, cheerful, lively; fatigue representing a negative affective state: fatigued, worn out, exhausted) rated on a 5-point scale ranging from *1—not at all* to *5—extremely*. We only used affect at t2 to examine the influence on subsequent physical activity. Both 3-item scales showed good internal consistencies on all 5 days (positive affect: α_day 2_ = 0.83 to α_day 4_ = 0.88; negative affect: α_day 3_ = 0.83 to α_day 5_ = 0.91).

#### Physical activity

##### Objectively measured moderate to vigorous physical activity

Physical activity was objectively measured using ActiGraph GT3X+ accelerometers (50 Hz, 60 s intervals). Participants were asked to wear the accelerometer on their right hip during waking hours except for water-related activities, such as swimming, bathing, or taking a shower (Swartz et al., 2000; Cain and Geremia, 2012). The average ActiGraph wear time was 14.9 h per day (ranging from 10.1 to 19.2 h), and all participants were included in the analysis (Ward et al., 2005; Cain and Geremia, 2012). Time spent in MVPA (minutes) were calculated as outcome variable based on 60 s intervals of aggregated counts with the software ActiLife (version 6.10.4). Choi's wear time criteria were used to validate wear time (Choi et al., 2011). To optimize data fidelity to our research question, person-specific daily filters were developed to extract physical activity data. The filters were based on the time points t2 and t3 when questionnaires were completed on weekdays. This time frame represents time after work. End times (t3) exceeding midnight, were replaced by 11:59 p.m. Because wear time after work varied between participants, we applied an additional 70/80 wear time validation for the extracted physical activity data (Catellier et al., 2005). Minimal wear time after work was defined as 262 min. Troiano's cut points were used to classify physical activity levels, thus ≥2020 counts-per-minute (CPM) indicated MVPA (Troiano et al., 2008).

##### Self-reported moderate to vigorous physical activity

Self-reported MVPA was operationalized via time spent with exercising and doing sports. This measure did not cover lifestyle activities such as active commuting. Every evening (t3), participants were asked to answer three questions regarding their MPVA during after work time on the smartphone. They reported how many minutes they had spent in MVPA [“How many minutes did you spent today exercising and doing sports? Please refer to those activities that raised your breathing rate and your heart rate and that made you sweat, e.g., exercising in the fitness studio, jogging, playing tennis, nordic-walking (but not light walking)”], the type of activity, and with whom they performed the activity.

#### Habit strength

The medium-term time-invariant variable habit strength was assessed at the beginning of the diary period using the German version (Thurn et al., 2014) of the Self-Report Habit Index (SRHI; Verplanken and Orbell, 2003). The SRHI is a frequently used scale for measuring habit strength by focusing on automaticity of the behavior (Gardner et al., 2011; Gardner, 2014). The stem “Behavior X is something…” (in this case: “Exercising is something…” is followed by 12 items (e.g., “…that I do without thinking”). The items were rated on a 4-point scale ranging from *0—“not true”* to *3—“true.”* The mean of the items represents habit strength with high scores indicating a strong habit. The items showed a good internal consistency in this study (α = 0.94).

### Data analysis

Multilevel regression models were used to analyze whether affect (day level) and habit strength (person level) predicted the objective after-work MVPA (outcome 1) and self-reported after work MVPA (outcome 2) using the Hierarchical Linear Modeling (HLM) Software version 7.01 (SSI, Inc., IL, USA). In addition, cross-level interactions (habit strength × positive/negative affect) were calculated to analyze whether habit strength had an impact on the influence of positive/negative affect on physical activity. Analyses were performed using random intercept models. Age and gender were included as covariates (person level). Day level variables were centered to the group mean and person level variables were included without centering. All variables were z-standardized to report standardized regression coefficients. For both physical activity outcome variables (objective and self-reported MVPA), separate models for positive and negative affect were calculated. This approach was chosen because of multi-collinearity as positive and negative affect were significantly correlated. Therefore, four models were calculated: positive affect and habit strength as predictors of objective MVPA (outcome 1, Model 1.1); negative affect and habit strength as predictors of objective MVPA (Model 1.2); positive affect and habit strength as predictors of self-reported MVPA (outcome 2, Model 2.1); negative affect and habit strength as predictors of self-reported MVPA (Model 2.2). These models were built up successively. The first step (M0) represents the zero-model to calculate the Intra-Class-Coefficient (ICC). In the second step (M1) the covariates (age and gender) were added to calculate the effects of the covariates. In the third step (M2) the day level variable affect was added. In the fourth step (M3) habit on the person level was added. In the last step (M4) the cross-level interaction was added.

## Results

Data of 30 men with a mean age of 43.8 years (*SD* = 10.8 years) and 59 women with a mean age of 45.2 years (*SD* = 8.1 years) were included in the analyses. Thirty-nine participants were full time and 44 were half time employed, one participant was on parental leave and four participants were homemakers. Fifty-six (62.6%) participants had a university-entrance diploma (“Abitur”) and six (6.6%) had an advanced technical college certificate (“Fachhochschulreife”). Seventy-four (83.1%) participants lived in a joint household with their spouse and 71 (79.8%) participants had at least one child living in the household.

### Descriptive results

On average, participants reported 31.2 min of MVPA (*SD* = 42.1 min, ICC = 0.22) during workdays. Eighty-five participants with 236 valid days of after work physical activity data were included in the analyses of objective after work MVPA. The average daily after work wear time was 353.9 min (*SD* = 79.1 min). On average, participants spent 4.9% (*SD* = 6.1%) of their after work time in MVPA representing 17.4 min per day (*SD* = 21.2 min). The portion of after work time spent in light physical activity was 33.1% (118 min), whereas 62.0% (218 min) of after work time was spent in sedentary activities. Self-reported and objective after work MVPA were only moderately correlated (*r* = 0.32, *p* < 0.001). The average reported positive affect was *M* = 3.49 (*SD* = 0.76, ICC = 0.38) and the average negative affect was *M* = 2.23 (*SD* = 0.87, ICC = 0.29). The ICC's showed that 62% (positive affect) up to 78% (self-reported MVPA) of the variance was caused by within-person variability. Habit strength was assessed at the beginning of the diary period. Participants reported a habit strength of regularly exercising of *M* = 1.83 (*SD* = 0.72).

### Prediction of objectively measured after work MVPA

The person level covariate age was positively related to objective after work MVPA (β = 0.18, *p* < 0.05; Table 1, M1). There was no association between gender and objective after work MVPA (β = −0.06, *p* = 0.46).

**Table 1:** **Model 1.1: Prediction of objective after work MVPA by positive affect and habit strength**.

**Day level**	**M0**	**M1**			**M2**			**M3**			**M4**		
	**ICC = 0.238**	**Beta**	***SE***	***p***	**Beta**	***SE***	***p***	**Beta**	***SE***	***p***	**Beta**	***SE***	***p***
Intercept		−0.025	0.078	0.748	−0.026	0.078	0.741	−0.012	0.074	0.870	−0.013	0.073	0.859
posA_t2					**0.231**	0.096	0.018	**0.231**	0.096	0.018	**0.264**	0.096	0.007
**PERSON LEVEL**													
habit								**0.230**	0.072	0.002	**0.230**	0.072	0.002
age		**0.182**	0.079	0.023	**0.182**	0.079	0.023	**0.179**	0.074	0.018	**0.179**	0.074	0.018
gender		−0.059	0.080	0.459	−0.059	0.079	0.461	−0.101	0.076	0.189	−0.100	0.076	0.190
**INTERACTION**													
habit x posA_t2											**0.205**	0.082	0.013
**Deviance**	654.43			655.23			654.26			646.23			645.01

Dependent variable: objective MVPA; posA_t2: positive affect measured at t2; habit: habit strength; covariates: age, gender; significant beta coefficients are indicated by bold numbers.

Positive affect, operationalized via three items representing vigor, significantly predicted objective after work MVPA (β = 0.23, *p* < 0.05; Table 1, M2). Higher positive affect in the afternoon was associated with more time subsequently spent in MVPA. There was a main effect of the time-invariant variable habit strength (β = 0.23, *p* < 0.01; Table 1, M3). Strong habits reported before the diary period were associated with higher levels of MVPA during the subsequent workdays. Moreover, there was a significant cross-level interaction effect (β = 0.21, *p* < 0.05) of positive affect (day level) and habit strength (person level; Table 1, M4). The effect of positive affect on after work MVPA differed depending on habit strength and was stronger in combination with a strong habit.

Subjective fatigue, representing a negative affective state, was negatively associated with time in after work MVPA (β = −0.20, *p* < 0.05), and the higher the negative affect the less MVPA was performed subsequently (Table 2, M2). Adding habit strength to the regression model revealed a significant main effect of habit strength (β = 0.23, *p* < 0.01; Table 2, M3). There was a significant cross-level interaction between habit strength and negative affect (β = −0.14, *p* < 0.05), and the association of negative affect and after work MVPA was influenced by habit strength (Table 2, M4). Contrary to our assumptions, habit strength seemed to strengthen the effect of negative affect.

**Table 2:** **Model 1.2: Prediction of objective after work MVPA by negative affect and habit strength**.

**Day level**	**M0**	**M1**			**M2**			**M3**			**M4**		
	**ICC = 0.238**	**Beta**	***SE***	***p***	**Beta**	***SE***	***p***	**Beta**	***SE***	***p***	**Beta**	***SE***	***p***
Intercept		−0.025	0.078	0.748	−0.003	0.078	0.741	−0.012	0.074	0.870	−0.013	0.073	0.864
negA_t2					−**0.203**	0.085	0.017	−**0.203**	0.084	0.017	−**0.244**	0.086	0.005
**PERSON LEVEL**													
habit								**0.230**	0.072	0.002	**0.230**	0.071	0.002
age		**0.182**	0.079	0.023	**0.182**	0.079	0.023	**0.179**	0.074	0.018	**0.179**	0.074	0.018
gender		−0.059	0.080	0.459	−0.059	0.079	0.461	−0.101	0.076	0.189	−0.101	0.076	0.189
**INTERACTION**													
habit x negA_t2											−**0.136**	0.069	0.048
**Deviance**	654.43			655.23			654.45			646.42			647.85

*Dependent variable: objective MVPA; negA_t2: negative affect measured at t2; habit: habit strength; covariates: age, gender; significant beta coefficients are indicated by bold numbers*.

Adding the cross-level interactions (M4) tended to increase the main effects of both positive (β = 0.26, *p* < 0.01) and negative affect (β = −0.24, *p* < 0.01) on objective after work MVPA (Tables 1, 2, M4).

### Prediction of self-reported after work MVPA

Neither age (β = 0.10, *p* = 0.16) nor gender (β = −0.05, *p* = 0.45) were significantly related to self-reported after work MVPA (Tables 3, 4, M1). The models revealed significant effects of positive (β = 0.18, *p* < 0.01) and negative affect (β = −0.13, *p* < 0.05; Tables 3, 4, M2) and habit strength (β = 0.28, *p* < 0.001) on after work MVPA (Tables 3, 4, M3). The cross-level interactions were not significant neither for positive (β = 0.01, *p* = 0.89) nor for negative affect (β = −0.01, *p* = 0.85; Tables 3, 4, M4).

**Table 3:** **Model 2.1: Prediction of self-reported after work MVPA by positive affect and habit strength**.

**Day level**	**M0**	**M1**			**M2**			**M3**			**M4**		
	**ICC = 0.223**	**Beta**	***SE***	***p***	**Beta**	***SE***	***p***	**Beta**	***SE***	***p***	**Beta**	***SE***	***p***
Intercept		−0.011	0.067	0.869	−0.003	0.068	0.969	−0.02	0.061	0.970	−0.002	0.060	0.969
posA_t2					**0.176**	0.058	0.002	**0.176**	0.058	0.002	**0.177**	0.070	0.012
**PERSON LEVEL**													
habit								**0.282**	0.062	< 0.001	**0.282**	0.059	< 0.001
age		0.096	0.067	0.155	0.084	0.068	0.223	0.082	0.062	0.187	0.082	0.077	0.290
gender		−0.051	0.067	0.446	−0.055	0.068	0.424	−0.098	0.062	0.117	−0.098	0.068	0.154
**INTERACTION**													
habit x posA_t2											0.009	0.064	0.890
**Deviance**	1219.23			1223.98			1205.31			1188.48			1194.10

*Dependent variable: self-reported MVPA; posA_t2: positive affect measured at t2; habit: habit strength; covariates: age, gender; significant beta coefficients are indicated by bold numbers*.

**Table 4:** **Model 2.2: Prediction of self-reported after work MVPA by negative affect and habit strength**.

**Day level**	**M0**	**M1**			**M2**			**M3**			**M4**		
	**ICC = 0.223**	**Beta**	***SE***	***p***	**Beta**	***SE***	***p***	**Beta**	***SE***	***p***	**Beta**	***SE***	***p***
Intercept		−0.011	0.067	0.869	−0.003	0.068	0.969	−0.002	0.061	0.970	−0.002	0.061	0.970
negA_t2					−**0.132**	0.055	0.017	−**0.132**	0.055	0.017	−**0.132**	0.055	0.017
**PERSON LEVEL**													
habit								**0.282**	0.062	< 0.001	**0.282**	0.062	< 0.001
age		0.096	0.067	0.155	0.084	0.068	0.223	0.082	0.062	0.187	**0.082**	0.062	0.187
gender		−0.051	0.067	0.446	−0.055	0.068	0.424	−0.098	0.062	0.117	−**0.098**	0.062	0.117
**INTERACTION**													
habit x negA_t2											−0.010	0.051	0.845
**Deviance**	1219.23			1223.98			1208.89			1192.06			1197.97

*Dependent variable: value self-reported MVPA; negA_t2: negative affect measured at t2; habit: habit strength; covariates: age, gender; significant beta coefficients are indicated by bold numbers*.

## Discussion

The aim of our study was to examine the effect of positive and negative affect on subsequent MVPA. In the last decade, the relationship between physical activity and affect received increasing attention, and there are two perspectives. First, it is assumed that exercising causes affective responses and these affective responses influence exercise adherence (Ekkekakis and Lind, 2006). While there is evidence for the benefits of physical activity on subsequent affective states (Kanning et al., 2013; Liao et al., 2015) only few studies have examined the link between affect and future subsequent physical activity (Williams, 2008).

Secondly, considering the important role of affects in regulating behavior (Russell, 2003; Baumeister et al., 2007) and the importance of behavior regulation in daily life, it seems obvious to assume that affective states impact subsequent physical activity. However, to date the pathway of the relationship between physical activity and affect has not been thoroughly investigated (Schwerdtfeger et al., 2010; Liao et al., 2015).

### Affect and subsequent MVPA

Consistent with preliminary evidence from other studies, we found that a positive affective state (operationalized as the feeling of vigor) in the afternoon was related to more time spent in MVPA in the subsequent hours. In agreement with our results, Dunton et al. (2009) showed that positive affect (operationalized via the rating of the adjective “happy”) was associated with higher levels of MVPA in the subsequent interval. Mata et al. (2012) explored the change in affect before engaging in physical activities and found that positive affect (operationalized via the rating of the adjectives “happy,” “excited,” “alert,” and “active”) increased over time before engaging in physical activities. However, this observation could originate from positive affect leading to engagement in physical activities or anticipating physical activity leading to increased positive affect. Carels et al. (2007) investigated a longer interval covering the entire day after affect assessment in the morning and showed that a positive affective state (measured via an one-item one-dimensional emotion scale) in the morning was related to the initiation of exercise during the day in obese adults, but not to its duration and intensity. However, it should be recognized that Dunton et al. (2009), Mata et al. (2012), and Carels et al. (2007) used self-report measures for both affective states and physical activity. Dunton et al. (2014) used accelerometers to measure MVPA and found that “feeling energetic” was associated with more time in MVPA in the subsequent 30 min in children. Schwerdtfeger et al. (2010) also used accelerometers and found that positive affect (operationalized via the rating of the adjectives “lively,” “awake,” “active,” “powerful,” “dynamic,” and “happy”) was significantly associated with higher levels of MVPA in four intervals: 1, 5, 15, and 30 min after affect measurement. In contrast to Schwerdtfeger et al. (2010) and Dunton et al. (2014), we considered a longer interval covering the time between answering the t2 questionnaire and going to bed. On average, this interval covered almost 6 h. Therefore, affect seems to influence subsequent short to medium term physical activity and this influence remains significant even when considering a longer interval of more than 5 h. Our study provides additional evidence regarding the role of positive affect in the regulation of physical activity behavior.

The results on negative affect were less consistent. In our study higher negative affect, operationalized via subjective fatigue, was associated with lower levels of subsequent MVPA. This result is consistent with the result of Dunton et al. (2009) (negative affect: “emotionally upset,” “stressed,” “lonely/alone,” “annoyed/angry,” “tense/anxious,” “sad/depressed,” and “discouraged/frustrated”) and Dunton et al. (2014) (emotional state: “feeling tired”) but in contrast to Schwerdtfeger et al. (2010) (“nervous,” “stressed,” “irritable,” “depressed,” “relaxed” '[-]), who reported increase in physical activity in the medium intervals (15 and 30 min) after assessment of negative affect. Mata et al. (2012) found no association between negative affect (“anxious,” “sad,” “disgusted,” “angry,” “guilty,” “ashamed,” “frustrated”) and subsequent physical activity. Hence, further research in this field in general is warranted, and especially the role of negative affect should be investigated in future studies.

Participants in our study overestimated their levels of MVPA by 45%. Nonetheless, we observed the same pattern of associations for objective and self-reported MVPA. However, the association between positive and negative affect and self-reported after work MVPA was weaker than that of objective after work MVPA. These slightly different results for objective and self-reported MVPA indicate that the link between affect and physical activity must be examined with a differentiated view on physical activity. Accelerometer and self-reported data reflect different facets of physical activity: while accelerometer data covers all bodily movements, self-reported physical activities refer to physical exercise and sports that are done mostly intentionally. Further studies should focus on different types of activity and on subsequent physical activity in different intervals.

Interestingly, the measurement of affect differed considerably among previous studies (Carels et al., 2007; Dunton et al., 2009, 2014; Schwerdtfeger et al., 2010; Mata et al., 2012) possibly explaining some of the inconsistencies in the results. For instance, Dunton et al. (2014) assessed positive affect via the adjectives “happy” and “joyful” and negative via the adjectives “stressed,” “mad or angry,” “nervous or anxious,” and “sad” (rated on a 4-point scale ranging from “not at all” to “extremely”) and found no associations with MVPA, whereas “physical feeling states,” operationalized via two items (“feeling tired” and “feeling energetic,” rated on the same 4-point scale) were significantly associated with subsequent MVPA. Feeling energetic was associated with higher levels of MVPA and feeling tired predicted lower levels of physical activity. In our study, negative affective state was operationalized via the feeling of fatigue measured with four items, and we found a negative association with physical activity. Whereas Schwerdtfeger et al. (2010) used the adjectives “nervous,” “stressed,” “irritable,” “depressed,” “relaxed(-)” and found an inverse relationship (negative affect predicted higher levels of physical activity). Mata et al. (2012) used the adjectives “anxious,” “sad,” “disgusted,” “angry,” “guilty,” “ashamed,” and “frustrated” and found no association. Moreover, it should be noted that different terms were used for equal constructs (for example mood, affect, affective states, feeling states). It is possible that the findings regarding the link between affect and physical activity differ depending on the facet of positive/negative affect that is measured, and maybe adjectives such as “happy” or “sad” are too general to be related to subsequent physical activity. Therefore, further studies should examine different facets of positive and negative affect and their association with subsequent MVPA.

Finally, previous studies have included different populations, and hence the observed inconsistencies may also be caused by population specific characteristics. Moreover, inter-individual differences in the link between affect and physical activity may be present because of individual characteristics.

### The role of habit strength

To the best of our knowledge, the role of time-invariant variables such as habit strength has not been investigated in relation to the affect-physical activity link. Habits are beneficial (although some may be disadvantageous, too) for regulating behavior in daily life because they require little cognitive effort and are efficient (Wood et al., 2002). Habitual behavior is less vulnerable to disturbances than deliberative behavior, and the stronger the habit to be physically active the more the persons are physically active (Gardner et al., 2011). As hypothesized we found that habit strength predicted after work MVPA throughout the week. In addition and in line with our assumption, there was a cross-level interaction effect for both positive and negative affect indicating that habit strength has an impact on the relationship between affect and behavior. However, in contrast to our hypothesis, the direction of this cross-level interaction indicated that a strong habit of being physically active strengthened the influence of negative affect. Negative affect reduced subsequent MVPA more in combination with a strong habit. It is possible that the previously mentioned benefit of cognitive efficacy and unconsciousness of habitual behavior turns into a disadvantage if self-control processes are not activated. Accordingly, we did not find a cross-level interaction effect for self-reported MVPA and the effect sizes of affect tended to be smaller. Self-reported MVPA mainly reflects intentional or planned physical activities representing the reflective pathway to action, and this pathway is characterized by conscious regulation processes. Negative affect seems to have a detrimental influence even on these physical activities but this effect was slightly smaller and was not strengthened in combination with a strong habit. Habit strength seems not to be relevant for the association between these activities and negative affect. Examining the role of intentions or self-regulatory processes in this context would be interesting. Moreover, studies on inter-individual differences in the link between affect and physical activity should integrate other person level variables, such as intrinsic vs. extrinsic motivation or goal orientation.

### Strength and limitations

A major strength of this study was the ambulatory assessment design allowing investigating the relationship between affect and physical activity between and within persons in daily life. We combined continuous accelerometer-monitoring of physical activity with electronic diary assessment of the momentary affective state. In addition, physical activity was measured subjectively via daily self-reports. Thus, the study design fully complies with the recommendations of Kanning et al. (2013) regarding the methodological requirements necessary for investigating within-subject associations between affective states and physical activity in daily life.

The fact that the sample was better educated than the average German population (Statistisches Bundesamt^2^) is a limitation of this study possibly limiting the generalizability of the findings. Moreover, 5 weekdays may not be a sufficient representation of usual behavior in daily life. However, longer electronic diary periods increase participants' burden and likely reduce compliance. We restricted affect measurement to eight items—four items for positive affect and four items for negative affect—to reduce participants' burden. Finally, although there appears to be a causal relationship between affect and subsequent MVPA, it is possible that other variables may influence both affect and physical activity in a similar way.

## Conclusion

In this study, we used ambulatory assessment to examine the relationship between affect and physical activity in a real-life setting. Positive affect increased and negative affect decreased objective and self-reported MVPA. Hence, affective states play an important role in the regulation of physical activity in daily life. Although more research is needed to confirm these findings, our results and those of few other studies indicate that considering unconscious processes, e.g., triggered via affects, might improve the explanation and prediction of engagement and especially maintenance of physical activity in daily life. Furthermore, adapting intervention strategies such as planning by considering the role of affects for daily behavior regulation, might improve long-term behavior change.

In addition, we found that the relationship between affect and physical activity is influenced by habit strength where the stronger the habit of being physically active the stronger the effect of positive but also of negative affect. Future studies should integrate time-invariant variables such as motives or goal orientations to gain further insight into the complexity of the relationship between affect and physical activity.

## Author contributions

CN was responsible for the overall conception, design and analysis of this study and wrote the manuscript. CH conducted the statistical analyses, contributed to the interpretation of the study results and revised the manuscript. BV and DV contributed to the conception of the manuscript, writing the manuscript, were responsible for preparing the accelerometer and electronic diary data for statistical analysis, the data management, and revised the manuscript. AW participated in the conception and design of the study. All authors read and approved the final manuscript.

## Funding

This study is part of the research project “EATMOTIVE” funded by the Federal Ministry of Education and Research, Germany (Grant 0315671).

### Conflict of interest statement

The authors declare that the research was conducted in the absence of any commercial or financial relationships that could be construed as a potential conflict of interest.

## Footnotes

^1^Deutsche Gesellschaft für Psychologie, Ethische Richtlinien der Deutschen Gesellschaft für Psychologie e.V. und des Berufsverbandes Deutscher Psychologinnen und Psychologen e.V. http://www.dgps.de/index.php?id=96422

^2^https://www.destatis.de/DE/ZahlenFakten/GesellschaftStaat/BildungForschungKultur/Bildungsstand/Tabellen/Bildungsabschluss.html

## Abbreviations

MVPA: moderate to vigorous physical activity.
